# The biarticularity of the gastrocnemii muscles provides relevant mechanisms for managing drop-like gait perturbations in humans

**DOI:** 10.1242/jeb.252394

**Published:** 2026-05-20

**Authors:** Christos Theodorakis, Sebastian Bohm, Falk Mersmann, Adamantios Arampatzis

**Affiliations:** ^1^ Department of Training and Movement Sciences, Humboldt-Universität zu Berlin, 10115 Berlin, Germany; ^2^ Berlin School of Movement Science, 10115 Berlin, Germany

**Keywords:** Uneven terrain, Energy transfer, Energy storage and recoil, Achilles tendon strain, Achilles tendon force, Triceps surae muscles

## Abstract

This study investigated the ankle-to-knee and knee-to-ankle joint energy transfer via the biarticular gastrocnemii muscles during unpredictable and adapted drop-like gait perturbations to understand how biarticular mechanisms of the gastrocnemii contribute to the mechanical work performed by the Achilles tendon (AT) force at the ankle joint. This was done by measuring AT elongation and quantifying AT force as an indicator of triceps surae muscle forces, as well as the body kinematics and electromyographic activity of the soleus, gastrocnemius medialis and gastrocnemius lateralis muscles, in 17 participants. Biarticular mechanisms contributed significantly to both the negative and positive mechanical work performed by the AT force at the ankle joint during both types of drop-like perturbations, constituting 17% to 26% of this mechanical work. In particular, during the initial stance phase of unpredictable, drop-like perturbations, a significant proportion of energy (26% of the negative mechanical work done at the ankle joint) was transferred from the ankle to the knee joint via the biarticular gastrocnemii muscles. More importantly, the rate of this energy transfer was elevated during the unpredictable perturbations, when beneficial stability control mechanisms based on prediction are unavailable, compared with adapted ones. Finally, our findings imply that elastic tissues contribute significantly to managing drop-like perturbations, including energy storage and recoil in the AT and potential for elastic energy exchange in the vasti tendons during the energy transfer phases. These findings could inform the design of prevention treatments and bioengineering approaches, especially for improving stability control in uneven terrain.

## Teaser

**Summary:** The rapid and substantial contribution of biarticular mechanisms to energy transfer between ankle and knee joints during perturbations facilitates the mechanical work performed at the ankle joint.

## INTRODUCTION

Both musculoskeletal model predictions and experimental studies have emphasized the significant contribution of biarticular mechanisms to perf­­ormance enhancements during various movement tasks (Arampatzis et al., 2023; Bobbert et al., 1986; Kharazi et al., 2023; Prilutsky and Zatsiorsky, 1994). The integration of biarticular mechanisms into bio-inspired robots, exoskeletons and prostheses has been considered a promising opportunity to make them more applicable to everyday situations (Chen et al., 2025; Schumacher et al., 2020; Wade et al., 2024). Given their capacity to influence two joints simultaneously, biarticular muscles can effectively target the joint that is more important for the desired movement (Cleland, 1867). They can also reduce the cost of neural control (Schumacher et al., 2020), enable adjacent monoarticular muscles to act at joints they do not span (Cleland, 1867; Van Ingen Schenau, 1989) and transfer mechanical energy between the larger proximal and the smaller distal leg muscles (Kharazi et al., 2023; Prilutsky and Zatsiorsky, 1994; Van Ingen Schenau, 1989). The advantages of muscle biarticularity for effective balance control across diverse movement conditions are supported by experimental studies (Schumacher et al., 2019), model predictions (Sharbafi et al., 2017) and robotic systems (Sharbafi et al., 2016). Recent studies indicate that while the involvement of the biarticular mechanisms of the gastrocnemii in the mechanical power and work performed at the ankle joint during level walking is minimal at slow or preferred speeds (2–5%; Kharazi et al., 2023), their contribution markedly increases during fast and maximal walking speeds, as well as during running (11–16%; Arampatzis et al., 2023; Kharazi et al., 2023), where the demand for mechanical power and work at the ankle joint is higher. These reports suggest a functional significance of biarticular muscles in locomotion within complex environments, involving movement on uneven terrain and gait perturbations (Arampatzis et al., 2025; Theodorakis et al., 2025).

It is widely accepted that the ankle joint plays an important role in both unperturbed and perturbed locomotion. The ankle joint accounts for 40–45% of the mechanical work and power required for walking, 58–67% for running (Arampatzis et al., 2000; Farris and Sawicki, 2012) and reaches 64% during slip-like perturbations (Golyski and Sawicki, 2022). Following drop-like perturbations, effective management of the centre of mass (CoM) energy is crucial for maintaining balance (Bohm et al., 2026; van Dieën et al., 2007). Rapid neuromuscular adjustments in kinematics, kinetics and activation patterns of the lower limbs were observed in animals including humans to absorb the elevated kinetic energy of the CoM during the subsequent stance phase (Bohm et al., 2026; Daley et al., 2006; Nakazawa et al., 2004; van Dieën et al., 2007). During drop-like perturbations, distal joints are important for the required kinetic energy absorption, with the ankle joint contributing between 66% and 80% (Dick et al., 2019), highlighting the significant role of the ankle joint in managing the body's CoM energy.

As the primary plantar flexors, the triceps surae muscles contribute critically to body stability control under demanding locomotor conditions (Bunz et al., 2023; Dick et al., 2021; Nakazawa et al., 2004). The soleus (SOL) muscle is monoarticular, the most voluminous and has the greatest physiological cross-sectional area (PCSA) among the triceps surae muscles (Albracht et al., 2008), generating the majority of the joint moment and power at the ankle joint during locomotion (Neptune et al., 2008; Pandy et al., 2010). The gastrocnemius medialis (GM) and gastrocnemius lateralis (GL) are biarticular muscles that span the ankle and knee joints, enabling them to transfer energy between the two joints (Bobbert et al., 1986; Kharazi et al., 2023). Recently, we observed a significant modulation of the energy transfer potential between the ankle and knee joints via the biarticular gastrocnemii muscles during drop-like perturbations (Theodorakis et al., 2025). The potential for energy transfer between the ankle and knee joints via the biarticular gastrocnemii muscles increased by a factor of 2.5 during drop-like gait perturbations compared with unperturbed level walking. This was accompanied by high gastrocnemii muscle activation during the phases of energy transfer, which indicates the relevance of biarticular mechanisms during the perturbations.

When the gastrocnemii muscles exhibit opposite mechanical power at the ankle and knee joints, this indicates energy transfer between the two joints (Arampatzis et al., 2023; Prilutsky et al., 1996). At the ankle joint, the gastrocnemii muscles generate plantarflexion moments, and at the knee joint they generate flexion moments. When dorsiflexion and knee flexion occur simultaneously, the mechanical power of the gastrocnemii muscles at the ankle and knee joints has opposite signs, indicating an energy transfer from the ankle to the knee joint (Arampatzis et al., 2023; Prilutsky et al., 1996). During synchronous knee extension and plantarflexion, the mechanical power of the gastrocnemii muscles also has opposite signs, indicating an energy transfer from the knee to the ankle joint (Arampatzis et al., 2023; Prilutsky et al., 1996). These possibilities for energy transfer between the ankle and knee joints, due to the biarticular nature of the gastrocnemii muscles, enable the more voluminous monoarticular vasti muscles (Mersmann et al., 2014, 2015) to contribute to mechanical power and work at the ankle joint. However, the extent of the transfer of energy from one joint to the other via the gastrocnemii muscles, and the contribution of the gastrocnemii's biarticular mechanisms to mechanical power and work at the ankle joint during drop-like gait perturbations, remains unknown.

The objective of the present study was to investigate the biarticular mechanisms of the gastrocnemii muscles during drop-like walking perturbations, given that walking constitutes the most common fall-related activity (∼47%; Li et al., 2006), with misplaced steps such as drop-like perturbations representing 12–20% of all fall cases (Berg et al., 1997; Boonkhao et al., 2024). The primary aim was to achieve a deeper understanding of how biarticular mechanisms of the gastrocnemii muscles contribute to the mechanical work performed by the Achilles tendon (AT) force at the ankle joint by quantifying the proportion of ankle-to-knee and knee-to-ankle joint energy transfer during unpredictable and adapted drop-like gait perturbations. We hypothesized that during drop-like perturbations, the proportion of energy transferred via the biarticular mechanisms of the gastrocnemii muscles between the ankle and knee joints contributes significantly to both the negative and positive mechanical work performed by the AT force at the ankle joint. Furthermore, we hypothesized that the rate of energy transfer from the ankle to the knee joint via the biarticular gastrocnemii muscles would be more pronounced during unpredictable drop-like perturbations, where predictive adjustments of motor control are not available.

## MATERIALS AND METHODS

### Participants and experimental design

**Fig. 1.**
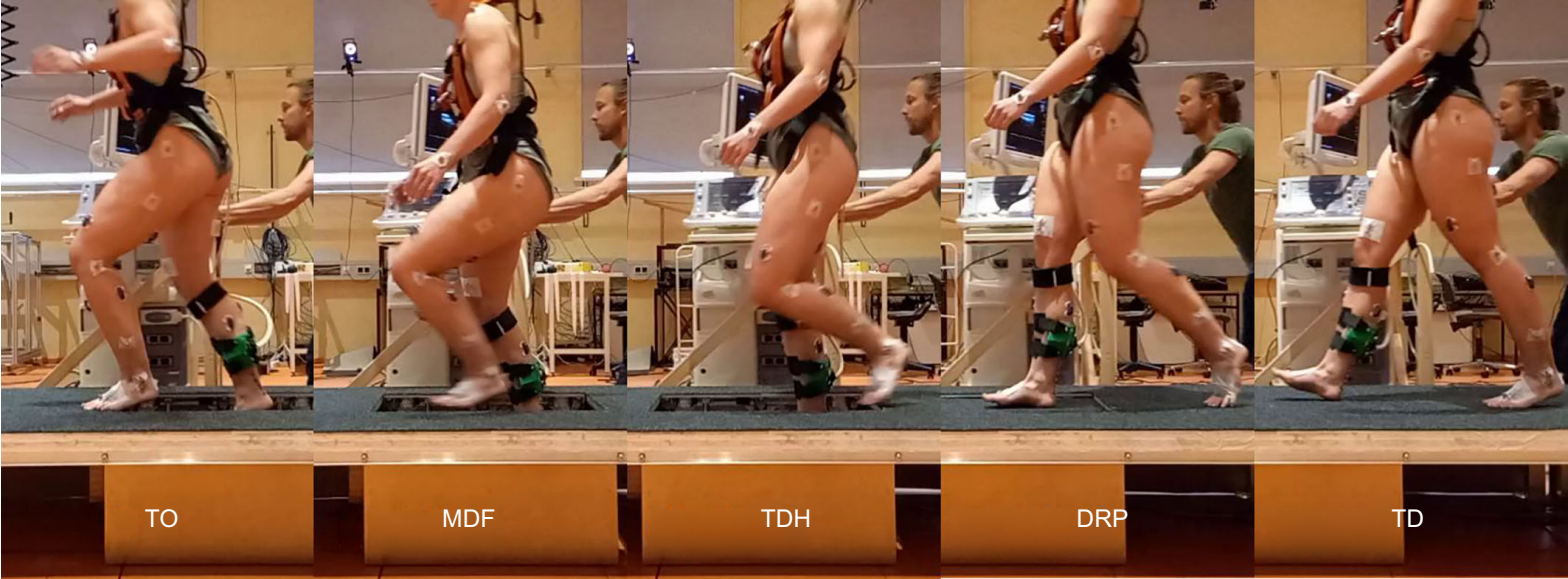
**Characteristic time points during the drop-like perturbations.** From right to left: touchdown of the perturbed leg before the plate drops (TD), drop of the plate (DRP), touchdown of the perturbed leg in the hole (TDH), maximum of dorsiflexion (MDF) and take-off of the perturbed leg (TO). Each participant completed three unperturbed walking trials, followed by one unpredictable drop-like perturbation and six predictable perturbations with prior announcement of the plate drop. The unpredictable and the sixth predictable (adapted) drop-like perturbations were analysed.

The trials were performed on an 18 m long and 1 m wide customized walkway with a hidden, electronically triggered drop-plate device (70×46×15 cm^3^) to induce unpredictable (without prior experience) and adapted (predictable with experience) drop-like gait perturbations at the participants' preferred walking speed (1.3±0.1 m s^−1^; Fig. 1). Approval for the experiment was granted by the ethics committee of Humboldt-Universität zu Berlin (HU-KSBF-EK_2022_0032). To determine the appropriate sample size, we performed an *a priori* power analysis (G*Power 3.1.9.4) using data from Bohm et al. (2026), in which the absorption of total CoM energy was higher during unpredictable drop-like perturbations compared with adapted ones, with a strong effect size (Cohen's *d*=0.83). With a power of at least 0.8 and an alpha level of 0.05, a sample size of 15 participants allows for the detection of statistically significant differences between the two perturbation conditions. Seventeen participants (5 female and 12 male, mean±s.d. age 24.7±5.8 years, body mass 75.4±10.2 kg, height 176.5±8.3 cm) without any acute or chronic neuromuscular impairments within the year prior to testing provided signed informed consent in accordance with the Declaration of Helsinki and were included in the study. Perturbations were applied at the beginning of the double support phase, approximately 30 ms after right foot strike. Participants completed three unperturbed walking trials, followed by one unpredictable and six predictable perturbation trials (with announced plate drop). Six repeated, predictable perturbations are sufficient to refine predictive adaptive responses (Bierbaum et al., 2010). The unpredictable and the sixth predictable perturbation trials were analysed to investigate reactive feedback responses in the absence of any anticipation of the perturbation, as well as the effects of experience-based predictive motor control. A harness system was used to avoid injury to participants in case of falling. As we found a very small effect in the energy transfer between the ankle and knee joints via the biarticular gastrocnemii muscles at the preferred walking speed (2–5% of the mechanical work performed by the AT at the ankle joint; Kharazi et al., 2023), we did not include unperturbed level walking in the current experiment.

### Body kinematics and electromyography

Whole-body kinematics were captured using a motion capture system of 19 cameras recording at 250 Hz (Vicon Nexus, version 2.12, Vicon Motion Systems, Oxford, UK). Data were recorded from 26 markers (14 mm diameter) attached to specific anatomically referenced locations: second metatarsal, tuber calcanei, lateral and medial malleoli, lateral and medial epicondyles, greater trochanter, acromion, elbow and wrist markers (bilaterally), sacrum, seventh cervical vertebra and cranial markers (two anterior and two posterior). The 3D marker coordinates were filtered using a fourth-order low-pass Butterworth filter with zero phase shift and a 12 Hz cut-off frequency. The touchdown and take-off of the foot were determined according to the method reported by Maiwald et al. (2009). In brief, the occurrence of a take-off is indicated by characteristic maximum of the vertical acceleration of the second metatarsal. Rear-foot touchdown is defined by the characteristic maximum of vertical acceleration at the calcaneus marker, while fore-foot touchdown is defined by the corresponding maximum at the second metatarsal marker. The ankle and knee joint angles were calculated in relation to a neutral, quiet stance body position. Positive values represent plantarflexed and knee-flexed (angle <180 deg) positions. Negative values at the ankle joint indicate dorsal flexion. Total CoM energy was calculated as the sum of potential and kinetic CoM energy (Eqn 1):
(1)

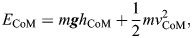
where *E*_CoM_ is total CoM energy (J), *m* is body mass (kg), ***g*** is acceleration of gravity (m s^−2^), *h*_CoM_ is CoM height (m) and *v*_CoM_ is CoM velocity vector (m s^−1^). The masses of the segments and their positions within each segment to calculate the CoM were taken from Dempster (1955), taking into account individual body mass and segment lengths.

The electromyographic (EMG) activity of the SOL, GM, GL vastus lateralis (VL) and vastus medialis (VM) muscles of the right (perturbed) leg was measured at 2000 Hz using a wireless EMG system (myon AG, Schwarzenberg, Switzerland), which was synchronized with the motion capture system. First, the raw EMG data were filtered using a fourth-order high-pass Butterworth filter with a 20 Hz cut-off frequency. Then, they were rectified and filtered using a low-pass filter with a 20 Hz cut-off frequency. The EMG data for the SOL, GM and GL were normalized to the maximum EMG activity obtained from each participant during a maximum voluntary fixed-end plantarflexion contraction (MVC). The VL and VM EMG data were normalized to the maximum achieved during fixed-end knee extension MVC. Finally, Zajac's first-order differential equation (Zajac, 1989) was used to assess the muscle activation (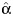) from the recorded normalized EMG activity (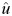) of the muscles (Eqn 2):
(2)

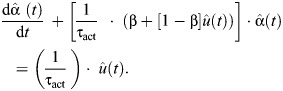
The specific activation time constant (τ_act_) and the ratio of activation to deactivation time constant (β) of slow and fast twitch fibres (Dick et al., 2017) were determined considering the reported fibre type distribution for SOL (slow: 78%, fast: 22%), GM, GL (slow: 50%, fast: 50%) and VL, VM (slow: 38%, fast: 62%) (Edgerton et al., 1975; Johnson et al., 1973).

### Quantification of AT force

**Fig. 2.**
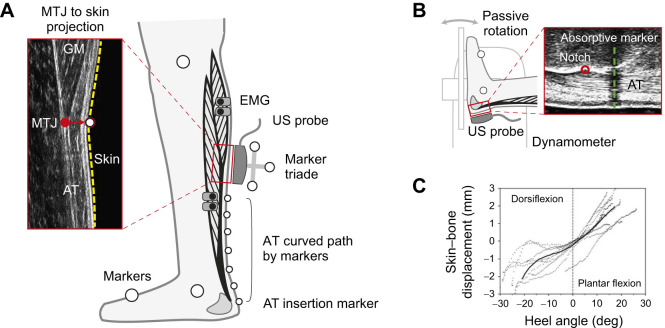
**Experimental setup for measuring Achilles tendon (AT) length during the drop-like perturbations.** (A) Reflective foil markers were applied to the skin to reconstruct the curved shape of the AT and to define the line of action of the AT. An ultrasound (US) probe was utilized to detect the movement of the gastrocnemius medialis myotendinous junction (MTJ) as the AT origin, which was subsequently projected onto the skin surface. Tripod markers were employed to transfer the detected positions of the MTJ to the global coordinate system. The AT lever arm was measured as the perpendicular distance from the rotation axis of the ankle joint (defined as the line connecting the malleoli) to the line of action of the AT. (B) To account for the relative skin displacement between the calcaneus marker and the notch of the tuber calcanei, a 3 cm US probe was positioned on the calcaneus bone, with a sound-absorbing marker placed between the probe and the bone. This setup enabled measurement of displacement differences between the calcaneus bone (reference point: notch) and the skin (marked by the absorber) as a function of the heel–shank angle. (C) Average (solid line) and individual (filled dots) data of skin-to-bone displacements versus heel angle.

To quantify the force of the AT, we measured AT elongation during the drop-like perturbations. The AT force was then determined based on the force–elongation relationship established for each participant during fixed-end plantar flexion MVCs, which were used as a calibration measure. The procedure for measuring AT length has been thoroughly documented in earlier work (Arampatzis et al., 2023; Kharazi et al., 2021, 2023). In brief, the curved path of the AT was measured using reflective markers placed on the skin (6 mm diameter), from the tuber calcanei notch (the AT's insertion point) to the most distal part of the GM myotendinous junction (the AT's origin point; Fig. 2). The GM myotendinous junction was registered using ultrasound (Aloka UST-5713T Hitachi Prosound Alpha 7, Hitachi, Japan) at a sampling frequency of 146 Hz. We used the motion capture system to measure the 3D coordinates of the skin reflective markers in the global space coordinate system. The GM myotendinous junction was tracked from the ultrasound images using a self-developed semi-automatic algorithm (Kharazi et al., 2021) implemented in MATLAB (version R2019a; The MathWorks Inc., Natick, MA, USA). The skin markers and GM myotendinous coordinates were filtered using a second-order, zero-phase shift Butterworth low-pass filter with a 6 Hz cut-off frequency. Afterwards, the coordinates of the GM myotendinous junction were projected onto the skin's surface within the ultrasound images. Three transformation matrices were applied to map the skin-projected coordinates to the global coordinate system. Initially, the coordinates derived from the ultrasound images were mapped to the probe's coordinate system, which was positioned on the probe's anterior protective surface and defined using a custom-fabricated 3D-printed calibration tool. Secondly, probe coordinates were mapped to a tripod-defined coordinate system mounted on the probe. In a different calibration procedure, using the motion capture system, we determined the position of the tripod relative to the ultrasound probe coordinate system and defined this second transformation matrix. Finally, the tripod coordinates were transformed to the global coordinate system using a transformation matrix defined by measuring the tripod's position with the motion capture system during the drop-like perturbations. Using the coordinates of the reflective markers on the skin and the transformed coordinates of the GM myotendinous junction to the global coordinate system, we determined the AT curved length as the sum of the Euclidean distances between consecutive points.

We also accounted for the relative skin displacement between the calcaneus marker and the notch of the tuber calcanei during all trials. We measured the displacement of a sound-absorbing skin marker relative to the notch of the tuber calcanei using a 3 cm ultrasound transducer (MyLab60, Esaote, Genoa, Italy; 37 Hz), when the foot was passively rotated by a dynamometer (Biodex System 3, Biodex Medical Systems Inc., Shirley, NY, USA) at 5 deg s^−1^ from 30 deg plantarflexion to maximum dorsiflexion, during three trials. The relative displacement of the absorptive skin marker was computed as a function of the heel angle – the angle formed by the line crossing the calcaneus marker and the midpoints of the lateral and medial malleoli and the shank – and served as a model to correct the AT length during the perturbations. The AT elongation was calculated as the difference between the AT length and the AT resting length, defined as the length of the curved path from the marker positioned at the notch of the tuber calcanei to the GM myotendinous junction. This measurement was taken in an inactive state at 20 deg plantarflexion, where force development within the muscle–tendon unit (MTU) is negligible or very low (De Monte et al., 2006). The AT strain was calculated as the quotient of the AT elongation and AT resting length. The instantaneous AT moment arm was determined as the perpendicular distance from the ankle's rotation axis, defined by the line connecting the lateral and medial malleoli markers, to the AT's line of action, incorporating the skin-to-tendon midpoint distance measured via sagittal plane ultrasound imaging.

We established the individual force–elongation relationship of the AT using five fixed-end plantarflexion MVCs on the Biodex dynamometer. We employed an established methodology to account for the influences of gravitational and passive forces, ankle–dynamometer axis misalignment, and effect of the inherent joint angular rotation on the myotendinous junction displacement during the MVCs (Arampatzis et al., 2005). The relevant kinematic data were acquired by the motion capture system at 250 Hz. Additionally, we used a well-established EMG-based approach to take into account the co-activation of the tibialis anterior muscle (Mademli et al., 2004). The AT force was calculated by dividing the active ankle joint moment by the instantaneous AT moment arm. A 10 cm linear ultrasound probe (MyLab60, Esaote; 25 Hz) was positioned above the GM myotendinous junction to measure AT elongation during the five MVCs. Sufficient reliability was achieved by averaging the AT force–elongation relationship across the five MVCs per participant (Schulze et al., 2012). The average AT force–elongation relationship was fitted using a quadratic function and served as a calibration measure to quantify AT force during the walking perturbations based on the measured AT elongation. The elastic strain energy of the AT was determined by integrating tendon force with respect to tendon elongation, while the energy rate was determined as the first-time derivative of tendon energy.

### Calculation of the triceps surae muscle kinetics

The distribution of AT force across the triceps surae muscles was calculated based on their PCSA fractions (SOL: 0.62, GM: 0.26, GL: 0.12; Albracht et al., 2008) and the determined activation profiles during the perturbations according to Eqn 3:
(3)

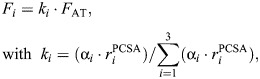
where *F_i_* is the force of muscle *i*, where *i* represents the SOL, GM and GL muscles; *F*_AT_ is AT force; *k_i_* is the distribution coefficient of muscle *i*; α*_i_* is the activation of muscle *i*; and 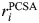 is the PCSA fraction of muscle *i*.

The mechanical power of the SOL, GM and GL at the ankle joint was calculated as in Eqn 4:
(4)

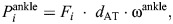
where 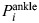 is mechanical power of muscle *i* at the ankle joint; *d*_AT_ is AT moment arm; and ω^ankle^ is angular velocity of the ankle joint.

The mechanical power of the GM and GL at the knee joint was calculated as in Eqn 5:
(5)

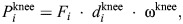
where 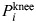 is mechanical power of muscle *i* at the knee joint; 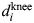 is moment arm of muscle *i* at the knee joint (GM and GL moment arms at the knee joint were extracted as a function of knee joint angle using the values reported by Buford et al., 1997); and ω^knee^ is angular velocity of the knee joint.

As a monoarticular muscle, the mechanical power of the SOL MTU is equal to its mechanical power at the ankle joint. The mechanical power of the biarticular GM MTU and GL MTU was calculated using Eqns 6 and 7 (Prilutsky et al., 1996):
(6)

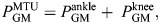

(7)

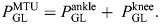


In our analysis, we considered the mechanical power of the gastrocnemii (GMGL) at the ankle and knee joints, as well as the mechanical power of their MTUs, by summing their respective power components.

Finally, we calculated the mechanical power of the triceps surae MTU and the mechanical power performed by the AT force at the ankle joint using Eqns 8 and 9:
(8)

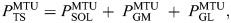

(9)

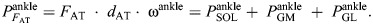


Mechanical work of the three muscles at the ankle and knee joints, as well as their MTUs, was subsequently calculated as the time integral of mechanical power. As noted in the Introduction, the mechanical power generated by the gastrocnemii muscles at the ankle and knee joints exhibits opposing signs during simultaneous dorsiflexion and knee flexion, i.e. negative values at the ankle joint and positive values at the knee joint, which implies inter-joint energy transfer from ankle to knee (Arampatzis et al., 2023; Prilutsky et al., 1996). The energy transferred from the ankle to the knee joint is quantified by integrating the positive mechanical power performed by the gastrocnemii at the knee joint during this phase. In contrast, during synchronous knee extension and plantarflexion, the mechanical power of the gastrocnemii muscles shows positive values at the ankle joint and negative values at the knee joint, i.e. opposite signs, indicating an energy transfer from the knee to the ankle joint (Arampatzis et al., 2023; Prilutsky et al., 1996). The energy transferred from the knee to the ankle joint is quantified by integrating the negative mechanical power performed by the gastrocnemii at the knee joint during this phase.

In our analysis, in addition to the primary variables of interest (i.e. energy transfer between ankle and knee joints), we examined the mechanical work of the SOL, GM and GL MTUs, as well as the mechanical work performed by the AT force at the ankle joint, to determine the contribution of biarticular mechanisms to ankle joint mechanical work. Additionally, variables such as ankle and knee joint angles and total CoM energy at touchdown in the hole after the plate drop are reported to examine predictive adjustments in adapted perturbations compared with unpredictable ones. Finally, to assess AT function as an elastic structure during drop-like perturbations, maximum AT force and strain as well as AT energy storage and recoil are reported.

### Sensitivity analysis

For the distribution of the AT force to the triceps surae muscles, we used the individual activation patterns of the three muscles for each participant. However, we utilized literature data on PCSA without accounting for the force–length–velocity relationships of the muscles. The length changes of the monoarticular SOL and the biarticular GM and GL may differ during the investigated stance phase, leading to differences in force–length and force–velocity potentials that were not considered in the calculation of muscle forces. We recently measured the fascicle length of the SOL muscle using ultrasonography during different locomotor tasks, including unpredictable and adapted drop-like perturbations similar to those in the current study (Bohm et al., 2026). We observed smaller changes in SOL fascicle length compared with the SOL MTU during the stance phase, with the SOL muscle exhibiting an average force–length–velocity potential of approximately 0.7. We performed a sensitivity analysis using the force–length–velocity potential of 0.7 for the SOL muscle, and modified the potentials of the GM and GL muscles in increments of 0.1 from 0.6 to 0.8 (nine combinations in total) to evaluate the effect of variable potentials on the primary outcomes of the study (i.e. the magnitude and rate of energy transferred between the ankle and knee joints). The new distribution coefficients used for the sensitivity analysis were calculated as in Eqn 10:
(10)

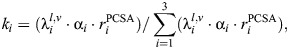
where 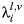 is the force–length–velocity potential of muscle *i*.

### Statistics

To evaluate the main effect of perturbation condition (unpredictable versus adapted), we employed a linear mixed model on the investigated outcomes: temporal parameters, joint kinematics, CoM and AT energy, AT strain and force, mechanical power, mechanical work and energy transfer (in total, 32 variables). In the applied linear mixed-effects model, participants are set as a random effect and perturbation condition is set as a fixed effect. According to the Shapiro–Wilk test applied to the normalized residuals, all investigated variables were found to be normally distributed. All statistical analyses were conducted in R version 4.0.1 (https://www.r-project.org/). A significance level of α=0.05 was selected for all tests, and Benjamini–Hochberg adjusted *P*-values for a total of 32 variables were reported. Additionally, Cohen's *d* was calculated to evaluate the magnitude of potential differences between the investigated variables. According to Cohen (1988), values of *d*<0.2 indicate small effect sizes, values of 0.2≤*d*<0.8 indicate medium effect sizes, and values of *d*≥0.8 suggest large effects.

## RESULTS

**Fig. 3.**
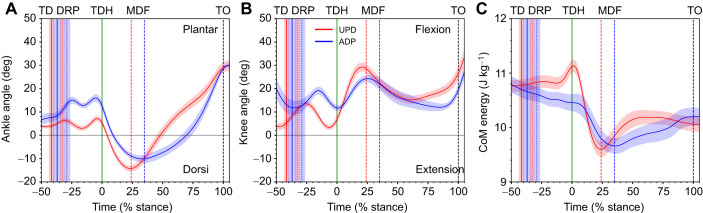
**Joint angles and total centre of mass (CoM) energy in the unpredictable (UPD) and adapted (ADP) drop-like gait perturbations.** (A) Ankle and (B) knee joint angle; (C) CoM energy. Positive values for the ankle angle represent a plantar flexed joint position and negative values represent a dorsiflexed joint position. The horizontal axis is normalized to the stance phase of the perturbed leg in the hole. The curves and shaded areas represent means±s.e.m. (*n*=17). TD, touchdown of the perturbed leg before the plate drops; DRP, drop of the plate; TDH: touchdown of the perturbed leg in the hole; MDF, maximum of dorsiflexion; TO, take-off of the perturbed leg.

The time from touchdown of the perturbed leg to plate drop was not significantly different between unpredictable and adapted perturbations (*P*=0.394, *d*=0.44) (Table 1). There was no difference in the duration of the stance phase between the two types of perturbation (*P*=0.471, *d*=0.27). At touchdown in the hole, the ankle joint was more plantarflexed (*P*=0.009, *d*=1.29) and the knee joint was more flexed (*P*=0.026, *d*=0.91) in the adapted perturbations (Table 1, Fig. 3A,B). The total CoM energy was higher at touchdown in the hole (*P*<0.001, *d*=1.19) in the unpredictable perturbations. In the first part of stance, the absorption of the total CoM energy was greater (*P*<0.001, *d*=1.99) and the duration of energy absorption was shorter (*P*<0.001, *d*=1.48) in the unpredictable perturbations (Table 1, Fig. 3C). There was no difference in the total CoM energy production or the duration of energy production during the second part of the stance phase between the two perturbations (*P*=0.511, *d*=0.23 and *P*=0.712, *d*=0.13; Table 1, Fig. 3C).

**Table 1.** Key spatiotemporal variables and Achilles tendon dynamics during drop-like perturbations

Variable	UPD	ADP
Time from TD to plate drop (ms)	40±10	36±9
Stance time (ms)	465±89	492±124
Ankle angle at TD in the hole (deg)*	5.3±4.7	12.6±6.5
Knee angle at TD in the hole (deg)*	6.9±5.7	11.7±4.6
Total CoM energy at TD in the hole (J kg^−1^)*	11.13±0.48	10.46±0.62
CoM energy absorption (J kg^−1^)*	−1.598±0.363	−0.899±0.348
CoM energy production (J kg^−1^)	0.535±0.449	0.642±0.393
Duration of CoM energy absorption (ms)*	105±17	147±36
Duration of CoM energy production (ms)	360±83	346±119
Maximum AT strain (%)	6.0±1.9	5.7±1.7
Maximum AT force (N kg^−1^)	52.8±18.7	50.1±22.6
Maximum AT energy storage and recoil (J kg^−1^)	0.251±0.152	0.231±0.127

Time from touchdown (TD) of the perturbed leg to plate drop, duration of the stance phase of the perturbed leg (stance time), ankle and knee joint angles at TD in the hole, total centre of mass (CoM) energy at TD in the hole, CoM energy absorption and production during the stance phase, duration of energy absorption and production, maximum Achilles tendon (AT) strain and force and maximum AT energy storage and recoil in the unpredictable (UPD) and adapted (ADP) drop-like gait perturbations. Means±s.d. (*n*=17). Asterisks indicate statistically significant differences (*P*<0.05).

**Fig. 4.**
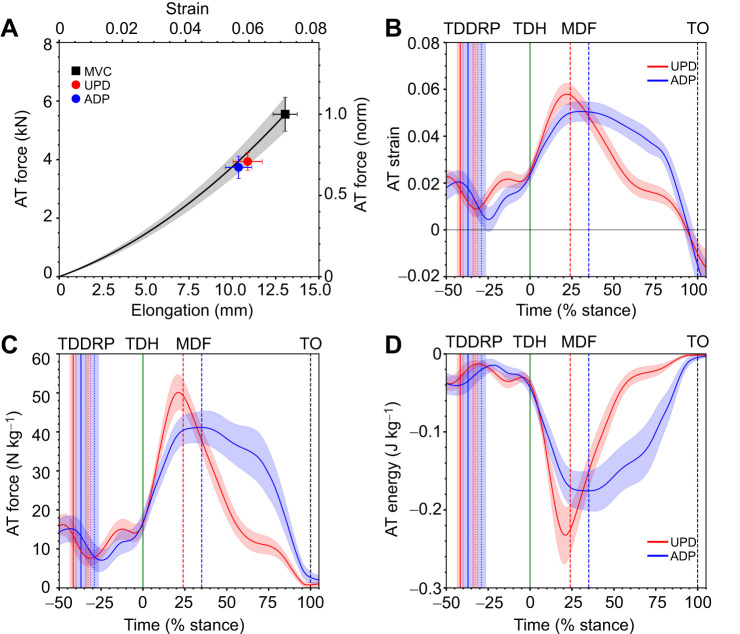
**AT force–elongation relationship and strain, force and energy over time during drop-like perturbations.** Force–elongation relationship of the AT during maximum voluntary fixed-end plantarflexion contractions (MVC) and the operating maximum AT force and strain values during the stance phase of the perturbed leg (A), AT strain (B), AT force (C) and AT energy (D) in the unpredictable (UPD) and adapted (ADP) drop-like gait perturbations. The horizontal axis is normalized to the stance phase of the perturbed leg in the hole. The curves and shaded areas represent means±s.e.m. (*n*=17). TD, touchdown of the perturbed leg before the plate drops; DRP, drop of the plate; TDH, touchdown of the perturbed leg in the hole; MDF, maximum of dorsiflexion; TO, take-off of the perturbed leg.

**Fig. 5.**
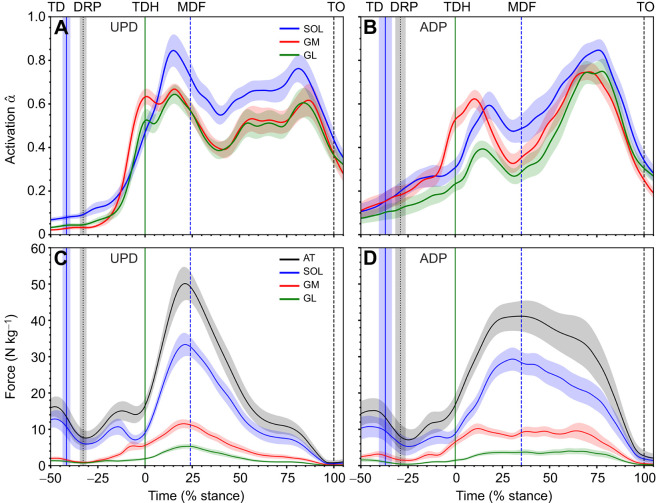
**Muscle activation patterns and AT and muscle forces during drop-like perturbations.** Activation patterns of the soleus (SOL), gastrocnemius medialis (GM) and gastrocnemius lateralis (GL) muscles (A,B) as well as forces of the AT, SOL, GM and GL muscles (C,D) in the unpredictable (UPD) and adapted (ADP) drop-like gait perturbations. The horizontal axis is normalized to the stance phase of the perturbed leg in the hole. The curves and shaded areas represent means±s.e.m. (*n*=17). TD, touchdown of the perturbed leg before the plate drops; DRP, drop of the plate; TDH, touchdown of the perturbed leg in the hole; MDF, maximum of dorsiflexion; TO, take-off of the perturbed leg.

During MVCs, the maximum applied force to the AT was 5551±1395 N, with the peak AT strain being 7.4±4.5%. The maximum AT strain and AT force showed no significant differences between the two types of perturbation (*P*=0.550, *d*=0.20 and *P*=0.712, *d*=0.14), with an operating maximum AT force of around 70% of that achieved during the MVCs (Table 1, Fig. 4A–C). The maximum AT energy storage and recoil did not differ between the two types of perturbations (*P*=0.698, *d*=0.15) (Table 1, Fig. 4D). The activation of the SOL, GM and GL muscles is characterized by two maxima, the first during dorsiflexion and the second during plantarflexion in both perturbations (Fig. 5A,B). In Fig. 5C,D, the time course of the SOL, GM and GL muscle forces during the unpredictable and adapted perturbations is also shown.

**Fig. 6.**
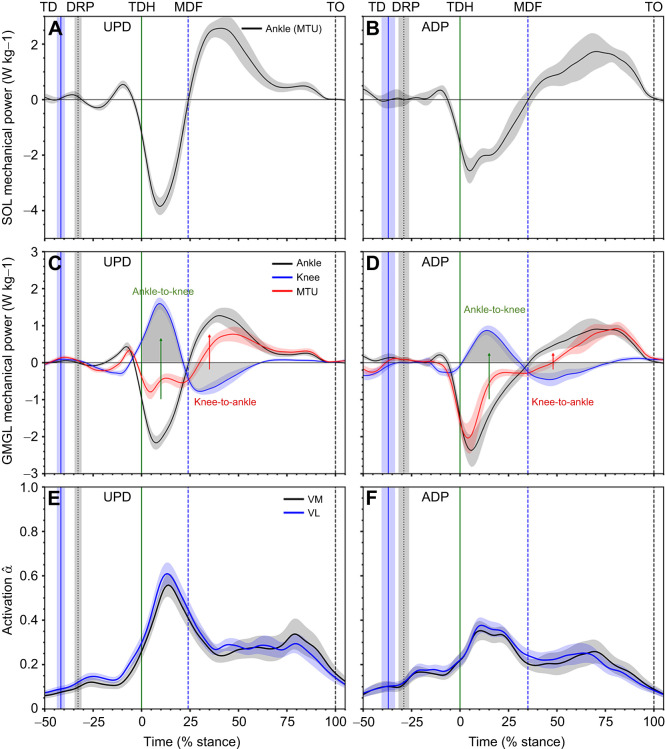
**Muscle mechanical power and activation during drop-like perturbations.** Mechanical power of the soleus (SOL) muscle at the ankle joint (A,B), mechanical power of the gastrocnemii (GMGL) muscles at the ankle and knee joints as well as mechanical power of the GMGL muscle–tendon unit (MTU) (C,D) and activation patterns of the vastus medialis (VM) and vastus lateralis (VL) muscles (E,F) in the unpredictable (UPD) and adapted (ADP) drop-like gait perturbations. The shaded areas in the mechanical power of the GMGL muscles at the knee joint illustrate the ankle-to-knee and knee-to-ankle joint energy transfer via their biarticularity. Note that the mechanical power of the monoarticular SOL muscle at the ankle joint is similar to that of the soleus MTU. The horizontal axis is normalized to the stance phase of the perturbed leg in the hole. The curves and shaded areas represent means±s.e.m. (*n*=17). TD, touchdown of the perturbed leg before the plate drops; DRP, drop of the plate; TDH, touchdown of the perturbed leg in the hole; MDF, maximum of dorsiflexion; TO, take-off of the perturbed leg.

**Fig. 7.**
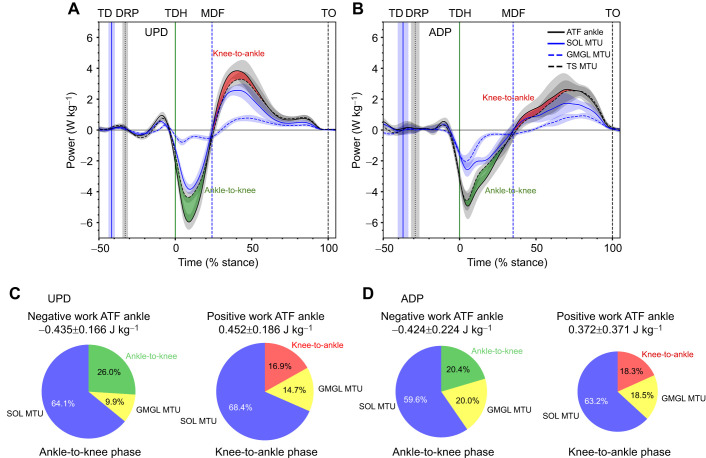
**Mechanical power of the AT and muscles during drop-like perturbations.** (A,B) Mechanical power of the AT force (ATF) at the ankle joint, mechanical power of the soleus muscle–tendon unit (SOL MTU), mechanical power of the gastrocnemii muscle–tendon unit (GMGL MTU) and mechanical power of the triceps surae muscle–tendon unit (TS MTU) in the (A) unpredictable (UPD) and (B) adapted (ADP) drop-like gait perturbations. The green and red areas represent the energy transfer from the ankle to the knee joint and from the knee to the ankle joint via the gastrocnemius muscles, respectively. The horizontal axis is normalized to the stance phase of the perturbed leg in the hole. The curves and shaded areas represent means±s.e.m. (*n*=17). TD, touchdown of the perturbed leg before the plate drops; DRP, drop of the plate; TDH, touchdown of the perturbed leg in the hole; MDF, maximum of dorsiflexion; TO, take-off of the perturbed leg. (C,D) Proportion of mechanical work done by the SOL MTU and GMGL MTU as well as energy transfer via the biarticular gastrocnemii muscles to the negative and positive mechanical work performed by the AT force at the ankle joint during the ankle-to-knee and knee-to-ankle energy transfer phases in the (C) UPD and (D) ADP drop-like gait perturbations. The size of each figure is scaled to the amount of the negative/positive mechanical work at the ankle joint.

The monoarticular SOL muscle performed negative mechanical power during the first part and positive mechanical power during the second part of the stance phase at the ankle joint (Fig. 6A,B). During the first part of stance, the synchronous dorsiflexion and knee flexion resulted in opposite signs of the mechanical power of the gastrocnemii at the two joints, thus transferring energy from the ankle to the knee joint via their biarticularity (Fig. 6C,D). During the second part of the stance phase, when the knee is extended and the ankle is plantarflexed, the gastrocnemii have again opposite signs of mechanical power at the two joints, thus transferring energy from the knee to the ankle via their biarticularity (Fig. 6C,D). During the phase of energy transfer from the ankle to the knee joint, the rate of energy transferred via the gastrocnemii muscles and the average mechanical power of the SOL MTU were significantly greater during unpredictable perturbations (*P*=0.014, *d*=1.02; *P*=0.027, *d*=1.02; Table 2 and Figs 6A–D and 7A,B). No other significant differences were observed in mechanical work, energy or power between the two types of perturbations during either energy transfer phase (*P*=0.107 to *P*=0.867, *d*=0.07 to *d*=0.66; Table 2).

**Table 2.** Key variables during the phases of energy transfer between the ankle and knee joints in the investigated drop-like perturbations

Variable	UPD	ADP
Anke-to-knee joint energy transfer phase		
SOL MTU work (J kg^−1^)	−0.276±0.097	−0.246±0.124
GMGL MTU work (J kg^−1^)	−0.046±0.050	−0.095±0.089
Ankle-to-knee energy transfer (J kg^−1^)	−0.113±0.059	−0.083±0.052
Average SOL MTU power (W kg^−1^) *	−3.06±98	−2.07±0.97
Average GMGL MTU power (W kg^−1^)	−0.52±0.56	−0.77±0.66
Rate of ankle-to-knee energy transfer (W kg^−1^) *	−1.22±0.55	−0.70±0.37
Knee-to-ankle joint energy transfer phase		
SOL MTU work (J kg^−1^)	0.302±0.108	0.245±0.236
GMGL MTU work (J kg^−1^)	0.072±0.060	0.067±0.082
Knee-to-ankle energy transfer (J kg^−1^)	0.077±0.050	0.060±0.061
Average SOL MTU power (W kg^−1^)	2.11±1.36	1.45±1.52
Average GMGL MTU power (W kg^−1^)	0.51±0.61	0.38±0.50
Rate of knee-to-ankle energy transfer (W kg^−1^)	0.56±0.52	0.37±0.42

Mechanical work of soleus (SOL) and gastrocnemii (GMGL) muscle–tendon unit (MTU), energy transfer from the ankle to the knee joint and vice versa via the gastrocnemii muscles, average mechanical power of SOL and GMGL MTU and rate of energy transfer from the ankle to the knee joint and vice versa during the ankle-to-knee and knee-to-ankle joint energy transfer phases in the unpredictable (UPD) and adapted (ADP) drop-like gait perturbations. Note that the mechanical work and power of the monoarticular SOL MTU is similar to that of the SOL at the ankle joint. Means±s.d. (*n*=17). Asterisks indicate statistically significant differences (*P*<0.05).

The proportion of mechanical work done by the GMGL MTU to the negative mechanical work performed by the AT force at the ankle joint during the ankle-to-knee energy transfer phase was significantly lower in the unpredictable compared with the adapted drop-like perturbations (9.9±8.5% versus 20.0±11.4%, *P*=0.014, *d*=1.02; Fig. 7C,D). On average, the proportion of ankle-to-knee energy transfer relative to the negative mechanical work done at the ankle joint was 26.0±9.5% for unpredictable drop-like perturbations and 20.4±6.9% for adapted ones, with no statistically significant differences between conditions (*P*=0.088, *d*=0.69; Fig. 7C,D). During the knee-to-ankle energy transfer phase, the proportions of SOL MTU, GMGL MTU and knee-to-ankle joint energy transfer relative to the positive mechanical work done at the ankle joint showed no significant differences between the two perturbation conditions (*P*≥0.214, *d*≤0.47; Fig. 7C,D).

As outcomes of the sensitivity analysis, we presented the lower and upper values of the proportion of ankle-to-knee and knee-to-ankle energy transfer to the negative and positive mechanical work performed at the ankle joint, respectively, as well as the rate of the ankle-to-knee joint energy transfer, which we found through the modification of the force–length–velocity potential of the SOL, GM and GL muscles (Table 3). Different force–length–velocity potentials affect the proportion of energy transferred between the ankle and knee joints; nevertheless, the proportion ranged from 18% to 28% for the ankle-to-knee joint energy transfer and from 15% to 19% for the knee-to-ankle joint energy transfer in all combinations (Table 3). Further, the rate of energy transferred from the ankle to the knee joint via the biarticular gastrocnemii remained significantly higher in the unpredictable drop-like perturbations compared with the adapted ones (all *P*≤0.022; Table 3).

**Table 3.** Key results of the applied sensitivity analysis

Variable	UPD	ADP
Proportion ankle-to-knee (%)		
Lower limit	23.5±7.8	17.9±5.7
Upper limit	28.1±9.6	21.3±6.9
Proportion knee-to-ankle (%)		
Lower limit	14.6±7.4	15.7±5.7
Upper limit	17.7±8.1	18.9±5.9
Rate of ankle-to-knee energy transfer (W kg^−1^)		
Lower limit*	−1.11±0.51	−0.63±0.34
Upper limit*	−1.32±0.6	−0.75±0.41

Range of the proportion of ankle-to-knee and knee-to-ankle joint energy transfer via the biarticular gastrocnemii muscles to the negative and positive mechanical work performed by the AT force at the ankle joint, respectively, as well as the rate of energy transfer from the ankle to the knee joint during the ankle-to-knee energy transfer phase in the unpredictable (UPD) and adapted (ADP) drop-like gait perturbations (results of the sensitivity analysis). Means±s.d. (*n*=17). Asterisks indicate statistically significant differences (*P*<0.05).

## DISCUSSION

For the first time, the modulation of energy transfer between the ankle and knee joints via the biarticular gastrocnemii muscles was examined during unpredictable and adapted drop-like gait perturbations. In both drop-like perturbations, we found that a significant proportion of the negative mechanical work performed by the AT force (i.e. 20–26%) is transferred from the ankle to the knee joint as a result of the biarticular mechanisms of the gastrocnemii muscles during the first part of the stance phase after the perturbation. During the second part of the stance phase, energy is transferred from the knee to the ankle joint via these biarticular mechanisms, accounting for 17–18% of the positive mechanical work performed by the AT force at the ankle joint. Furthermore, we observed a higher rate of energy transfer from the ankle to the knee joint via the gastrocnemii muscles during unpredictable drop-like perturbations compared with adapted perturbations, with this difference showing a large effect size. In particular, during the initial stance phase of unpredictable, drop-like perturbations when beneficial stability control mechanisms based on prediction are unavailable, the rate of ankle-to-knee joint energy transfer was 74% higher compared with the adapted ones. The applied sensitivity analysis revealed that these findings remained valid with respect to the variation of the force–length–velocity potentials of the SOL, GM and GL muscles, and thus with the force sharing among the triceps surae muscles. Our results thus supported both defined hypotheses. The SOL muscle, as the most voluminous of the triceps surae muscles (Albracht et al., 2008; Mersmann et al., 2014), accounted for 64.1% of the energy absorbed at the ankle joint, demonstrating its crucial role in managing drop-like perturbations.

During the stance phase, the maximum AT force was approximately 70% of MVC, being significantly higher than the values observed during level walking (40% MVC; Kharazi et al., 2023) and running (52% MVC; Arampatzis et al., 2023). On average, the musculoskeletal system absorbed 120 J of the total CoM energy following an unpredictable perturbation and 68 J following an adapted perturbation in the first part of the stance phase. The negative mechanical work performed by the AT force at the ankle joint was 27.2% and 47.2% of the total CoM energy absorption during unpredictable and adapted perturbations, respectively, showing the importance of this joint. During the unpredictable drop-like perturbations, the body's CoM energy is absorbed within the first 105 ms after touchdown in the hole. Because of the unpredictable nature of the applied perturbations and the lack of experience with them, it is not possible to make predictive adjustments before the plate drops (Van Der Linden et al., 2007). Furthermore, if present at all, the contribution of supraspinal neural control for corrective responses during the first 105 ms after touchdown in the hole is negligible (Jakubowski et al., 2025). Conversely, the biarticular mechanisms of the gastrocnemii muscles, as an entity of the musculoskeletal system's structure, can be involved automatically and respond rapidly to unpredictable drop-like perturbations by transferring energy from the ankle to the knee joint. This energy transfer enhances the negative mechanical work done at the ankle joint, reduces the need for time-consuming targeted cortical neural processes and assists in managing the body's CoM energy. During this brief period of 105 ms, 26% of the negative mechanical work performed by the AT force at the ankle joint was transferred to the knee via the biarticular gastrocnemii muscles. Although the amount of energy transferred from the ankle to the knee joint did not differ between the two types of perturbation, the higher rate of energy transfer in the unpredictable perturbations demonstrates the rapid and efficient activation of biarticular mechanisms for energy absorption following unpredictable drop-like gait perturbations.

The plate dropped 36 to 40 ms after touchdown of the perturbed leg, with no difference between the two perturbations, and was triggered during the double contact phase in all participants. During unpredictable perturbations, the activation of all three triceps surae muscles increased in a reactive manner after the plate was dropped, achieving maximum values during the ankle-to-knee energy transfer phase. The fast reactive increase in activation of the triceps surae muscles resulted in a rapid increase in muscle and AT forces during the initial stance phase. This increase in muscle activation and consequently in muscle forces was the reason for the fast involvement of biarticular mechanisms and for the increased rate of energy transfer from ankle to knee joint, in response to the unpredictable drop-like perturbations. In a recent study investigating the energy transfer potential and activation of the gastrocnemii muscles during unpredictable and adapted drop-like perturbations, we found that the ankle-to-knee joint energy transfer potential was similar in the two types of perturbation (Theodorakis et al., 2025). However, there was higher gastrocnemii muscle activation during the ankle-to-knee energy transfer phase in the unpredictable perturbations (Theodorakis et al., 2025). Therefore, we suggested that biarticular mechanisms may play a greater functional role in unpredictable perturbations than in adapted ones (Theodorakis et al., 2025). In the current study, we confirmed this suggestion and demonstrated that the increased rate of energy transfer from the ankle to the knee joint during the critical initial stance phase in response to unpredictable perturbations constitutes a key feature of gastrocnemii biarticularity for energy absorption.

In line with previous reports, predictive adjustments were initiated during the adapted perturbations to improve the management of the expected perturbation. The more plantarflexed ankle and more flexed knee joint at touchdown in the hole are important leg adjustments that facilitate energy absorption (Van Der Linden et al., 2007) and moment generation (Herzog et al., 1990). The reduced CoM energy at touchdown during the adapted perturbations results from proactive CoM lowering, which enables the contralateral leg to absorb energy (Arampatzis et al., 2025; Van Der Linden et al., 2007). These predictive adjustments decreased the magnitude and extended the duration of the body's CoM energy absorption compared with unpredictable drop-like perturbations, thereby making energy management less challenging during the adapted drop-like perturbations. As a consequence, the rate of energy transfer from the ankle to the knee joint and the mechanical power of the SOL MTU were also reduced in the adapted perturbations.

In the second part of the stance, i.e. from approximately 25% to 60% of stance for unpredictable and 35% to 80% of stance for adapted perturbations, energy is transferred from the knee to the ankle joint via the biarticular gastrocnemii muscles. During the knee-to-ankle energy transfer phase, the proportion of energy transferred from the knee to the ankle joint constituted 16.9% of the positive mechanical work performed by the AT force at the ankle joint for unpredictable perturbations and 18.3% for adapted perturbations. In both types of perturbation, the knee-to-ankle energy transfer during the push-off phase increases the positive mechanical power of the AT force at the ankle joint. We observed a similar phenomenon, i.e. an enhancement in mechanical power at the ankle joint during the push-off phase, which was attributed to the biarticular mechanisms of the gastrocnemii muscles during fast and maximum walking speeds (Kharazi et al., 2023) and during running (Arampatzis et al., 2023). Musculoskeletal models also predict an increase in mechanical power at the ankle joint during maximum vertical jumping and accelerated sprinting, via an energy transfer from the knee to the ankle joint through the gastrocnemii muscles (Bobbert et al., 1986; Jacobs et al., 1996; Prilutsky and Zatsiorsky, 1994). Different types of perturbations, including drop-like ones, require rapid motor responses that are functionally relevant for maintaining body stability and preventing falls (Bierbaum et al., 2011; Pijnappels et al., 2005; Van Der Linden et al., 2007). Recently, we found that the potential for energy transfer from the knee to the ankle joint through the biarticular gastrocnemii muscles increases during trip-like and drop-like gait perturbations compared with level walking (Theodorakis et al., 2025), and that this potential also increases with running speed from slow to maximum (Bohm et al., 2025). The above findings indicate that rapid motor responses which are critical for safety and performance during demanding locomotor tasks lead to a greater utilization of biarticular mechanisms.

The efficiency of energy transfer mechanisms between the ankle and knee joints due to the biarticular nature of the gastrocnemii muscles lies in the principle that energy can be absorbed and produced by the large, monoarticular vasti muscles, which can be utilized efficiently at the ankle joint (Van Ingen Schenau, 1989). The forces generated by the gastrocnemii muscles produce flexion moments at the knee joint, which must be counteracted by the knee extensors. The monoarticular vasti, as the largest knee extensor muscle group and being 5.7 times larger than the rectus femoris (Handsfield et al., 2014), counteract the majority of these gastrocnemii-induced knee flexion moments. In both perturbations, the vasti muscles were active during the ankle-to-knee and knee-to-ankle energy transfer phases (33–50% and 24–29% MVC, respectively), indicating force generation by these muscles. During the ankle-to-knee joint energy transfer phase, knee flexion results in active lengthening of the vasti-MTUs. This active lengthening suggests that the majority of energy transferred from the ankle to the knee joint is absorbed by the vasti MTUs, as they are much more voluminous than the rectus femoris. Regardless of whether the contractile elements of the vasti muscles absorb energy during this phase, some of the transferred energy will be stored as elastic energy in their tendons. During the subsequent knee-to-ankle energy transfer phase, the knee joint extends and the vasti-MTUs actively shorten, indicating energy production. Part of this energy originates from the elastic energy stored in the vasti tendons, which was previously transferred from the ankle to the knee joint through the gastrocnemii muscles. Therefore, it can be argued that the involvement of biarticular mechanisms of the gastrocnemii muscles in managing drop-like perturbations includes an energy exchange between elastic tissues. Furthermore, substantial energy was stored and recoiled in the AT during both perturbations. On average, the maximum AT energy storage and recoil accounted for 15.7% of the absorbed CoM energy during unpredictable perturbations and 25.6% during adapted ones, thus demonstrating significant elastic energy exchange and functional importance in such gait perturbations. Transient tendon energy storage as a buffering mechanism is crucial for effective movement in animals (Konow et al., 2012; Roberts and Konow, 2013) including humans (Arampatzis et al., 2023; Werkhausen et al., 2017).

In our experimental design, we informed the participants that something would be introduced while they were walking, which would disrupt their gait pattern. Consequently, the perturbations were not entirely unexpected. This approach was adopted for ethical reasons, which is why the term ‘unpredictable drop-like perturbations’ was used throughout the paper. Nevertheless, the participants were unaware of the type of perturbation and when it would occur, and had no prior experience of the induced, drop-like perturbations. AT force was quantified using the AT force–elongation relationship derived from MVCs as a calibration measure. Although the triceps surae muscles generate the majority of the ankle joint moment during plantarflexion, other plantar flexor muscles also contribute to this active moment. However, the PCSA of the triceps surae muscles constitutes approximately 80% of the total PCSA of all plantar flexors (Fukunaga et al., 1996). Furthermore, the AT moment arm is approximately 3.7 times larger than those of other plantar flexor muscles (Klein et al., 1996; Spoor et al., 1990). This implies that the aforementioned limitation has negligible influence on the conclusions regarding the contribution of biarticular gastrocnemii mechanisms to mechanical power and work at the ankle joint during drop-like perturbations. Given the viscoelastic nature of tendons as biomaterials, the rate of AT force development may impact the precision of force quantification. The AT force–elongation relationship, used as a calibration measure, was established during MVCs with a rate of force development of 1.3±0.6 kN s^−1^, considerably lower than that during the unpredictable (25.9±15.5 kN s^−1^) and adapted (14.7±9.1 kN s^−1^) perturbations. Ker (1981) has reported that loading rates from 0.6 to 31.2 kN s^−1^ do not affect the tendon's Young's modulus; thus, it can be argued that loading rate has a negligible effect on tendon dynamics during drop-like perturbations.

This investigation is the first to document how biarticular mechanisms of the gastrocnemii muscles are involved in managing drop-like perturbations, a challenging locomotor task, by quantifying the proportion of ankle-to-knee and knee-to-ankle joint energy transfer to the mechanical work performed by the AT force at the ankle joint. We found that the biarticular mechanisms of the gastrocnemii muscles make an important contribution in the mechanical work required at the ankle joint during both unpredictable and adapted drop-like perturbations. The significant and rapid contribution of biarticular mechanisms to the negative mechanical work at the ankle joint during the first 105 ms of the stance phase, particularly following unpredictable drop-like perturbations, highlights the importance of these mechanisms in managing such perturbations. Taking into account the energy storage and recoil in the AT, as well as the possible energy storage and recoil in the vasti tendons during the ankle-to-knee and knee-to-ankle energy transfer phases, our findings indicate that elastic tissues contributed to body energy regulation during drop-like perturbations. These findings could be important for the conceptualization of prevention and rehabilitation treatments that aim to improve stability control in healthy and pathological individuals. For example, exercises designed to elicit in-phase fluctuations between ankle and knee joints may enhance preventive and rehabilitative interventions by teaching individuals to utilize biarticular mechanisms quickly. The results could also help to improve the functionality of prostheses, exoskeletons and bipedal robots by enabling stable movement on uneven terrain, particularly when biarticular mechanisms and tissue elasticity are incorporated into their design.

## References

[JEB252394C1] Albracht, K., Arampatzis, A. and Baltzopoulos, V. (2008). Assessment of muscle volume and physiological cross-sectional area of the human triceps surae muscle in vivo. *J. Biomech.* 41, 2211-2218. 10.1016/j.jbiomech.2008.04.020 18555257

[JEB252394C2] Arampatzis, A., Knicker, A., Metzler, V. and Brüggemann, G. P. (2000). Mechanical power in running: a comparison of different approaches. *J. Biomech.* 33, 457-463. 10.1016/S0021-9290(99)00187-6 10768394

[JEB252394C3] Arampatzis, A., Morey-Klapsing, G., Karamanidis, K., DeMonte, G., Stafilidis, S. and Brüggemann, G.-P. (2005). Differences between measured and resultant joint moments during isometric contractions at the ankle joint. *J. Biomech.* 38, 885-892. 10.1016/j.jbiomech.2004.04.027 15713310

[JEB252394C4] Arampatzis, A., Kharazi, M., Theodorakis, C., Mersmann, F. and Bohm, S. (2023). Biarticular mechanisms of the gastrocnemii muscles enhance ankle mechanical power and work during running. *R. Soc. Open Sci.* 10, 230007. 10.1098/rsos.230007 37650058 PMC10465202

[JEB252394C5] Arampatzis, A., Nikolaidou, M.-E., Theodorakis, C., Ghasemi, M., Mersmann, F. and Bohm, S. (2025). Functional trade-offs of regulatory mechanisms in the management of body energy, frontal plane angular momentum and mediolateral margin of stability during hole negotiation gait. *Ann. Biomed. Eng.* 53, 3126-3140. 10.1007/s10439-025-03835-7 40921867 PMC12575527

[JEB252394C6] Berg, W. P., Alessio, H. M., Mills, E. M. and Tong, C. (1997). Circumstances and consequences of falls in independent community-dwelling older adults. *Age Ageing* 26, 261-268. 10.1093/ageing/26.4.261 9271288

[JEB252394C7] Bierbaum, S., Peper, A., Karamanidis, K. and Arampatzis, A. (2010). Adaptational responses in dynamic stability during disturbed walking in the elderly. *J. Biomech.* 43, 2362-2368. 10.1016/j.jbiomech.2010.04.025 20472240

[JEB252394C8] Bierbaum, S., Peper, A., Karamanidis, K. and Arampatzis, A. (2011). Adaptive feedback potential in dynamic stability during disturbed walking in the elderly. *J. Biomech.* 44, 1921-1926. 10.1016/j.jbiomech.2011.04.027 21555126

[JEB252394C9] Bobbert, M. F., Mackay, M., Schinkelshoek, D., Huijing, P. A. and van Ingen Schenau, G. J. (1986). Biomechanical analysis of drop and countermovement jumps. *Eur. J. Appl. Physiol.* 54, 566-573. 10.1007/BF00943342 3948851

[JEB252394C10] Bohm, S., Theodorakis, C., Ghasemi, M., Mersmann, F. and Arampatzis, A. (2025). Biarticular gastrocnemii muscles increase their joint energy transfer potential at high running speeds. *R. Soc. Open Sci.* 12, 241933. 10.1098/rsos.241933 40271140 PMC12014234

[JEB252394C11] Bohm, S., Ghasemi, M., Theodorakis, C., Mersmann, F., Roberts, T. and Arampatzis, A. (2026). Soleus muscle stiffness is regulated by scaled activation to manage unpredictable and predictable walking perturbations. *Ann. Biomed. Eng.* 54, 750-766. 10.1007/s10439-025-03928-3 41388230 PMC12960456

[JEB252394C12] Boonkhao, L., Puangjan, K., Ouengprasert, I., Laosupap, K., Bootsorn, A., Junsiri, S., Thongdamrongtham, S., Chaikhan, S., Pramaya, P. and Rattanachaikunsopon, P. (2024). Home environmental factors associated with falls among elderly in Ubon Ratchathani, Thailand. *J. Multidiscip. Healthc.* 17, 1363-1373. 10.2147/JMDH.S456128 38560486 PMC10981419

[JEB252394C13] Buford, W. L., Ivey, F. M., Malone, J. D., Patterson, R. M., Peare, G. L., Nguyen, D. K. and Stewart, A. A. (1997). Muscle balance at the knee--moment arms for the normal knee and the ACL-minus knee. *IEEE Trans. Rehabil. Eng. Publ. IEEE Eng. Med. Biol. Soc.* 5, 367-379. 10.1109/86.650292 9422462

[JEB252394C14] Bunz, E. K., Haeufle, D. F. B., Remy, C. D. and Schmitt, S. (2023). Bioinspired preactivation reflex increases robustness of walking on rough terrain. *Sci. Rep.* 13, 13219. 10.1038/s41598-023-39364-3 37580375 PMC10425464

[JEB252394C15] Chen, T., Liu, Z., Li, C., Chen, X., Hu, J. and He, Y. (2025). Enhanced gastrocnemius-mimicking lower limb powered exoskeleton robot. *J. NeuroEngineering Rehabil.* 22, 175. 10.1186/s12984-025-01703-y PMC12323188 40760664

[JEB252394C16] Cleland, J. (1867). On the actions of muscles passing over more than one joint. *J. Anat. Physiol.* 1, 85-93. PMC1318532 17230710

[JEB252394C17] Cohen, J. (1988). *Statistical Power Analysis for the Behavioral Sciences*, 2nd edn New York: Routledge.

[JEB252394C18] Daley, M. A., Voloshina, A., Biewener, A. A., Daley, M. A., Usherwood, J. R., Felix, G. and Biewener, A. A. (2006). Unsteady Locomotion - integrating muscle function with whole body mechanics. *J. Exp. Biol.* 210, 2949-2960. 10.1242/jeb.005801 PMC2651961 17704070

[JEB252394C19] De Monte, G., Arampatzis, A., Stogiannari, C. and Karamanidis, K. (2006). In vivo motion transmission in the inactive gastrocnemius medialis muscle-tendon unit during ankle and knee joint rotation. *J. Electromyogr. Kinesiol. Off. J. Int. Soc. Electrophysiol. Kinesiol.* 16, 413-422. 10.1016/j.jelekin.2005.10.001 16309922

[JEB252394C20] Dempster, W. T. (1955). *Space requirements of the seated operator: geometrical, kinematic, and mechanical aspects of the body, with special reference to the limbs*. *Wright Air Development Center Technical Report 55-159*. Wright-Patterson Air Force Base, Ohio: Wright Air Development Center, Air Research and Development Command, United States Air Force.

[JEB252394C21] Dick, T. J. M., Biewener, A. A. and Wakeling, J. M. (2017). Comparison of human gastrocnemius forces predicted by Hill-type muscle models and estimated from ultrasound images. *J. Exp. Biol.* 220, 1643-1653. 10.1242/jeb.154807 28202584 PMC5450802

[JEB252394C22] Dick, T. J. M., Punith, L. K. and Sawicki, G. S. (2019). Humans falling in holes: adaptations in lower-limb joint mechanics in response to a rapid change in substrate height during human hopping. *J. R. Soc. Interface* 16, 20190292. 10.1098/rsif.2019.0292 31575349 PMC6833322

[JEB252394C23] Dick, T. J. M., Clemente, C. J., Punith, L. K. and Sawicki, G. S. (2021). Series elasticity facilitates safe plantar flexor muscle–tendon shock absorption during perturbed human hopping. *Proc. R. Soc. B Biol. Sci.* 288, 20210201. 10.1098/rspb.2021.0201 PMC8059679 33726594

[JEB252394C24] Edgerton, V. R., Smith, J. L. and Simpson, D. R. (1975). Muscle fibre type populations of human leg muscles. *Histochem. J.* 7, 259-266. 10.1007/BF01003594 123895

[JEB252394C25] Farris, D. J. and Sawicki, G. S. (2012). The mechanics and energetics of human walking and running: a joint level perspective. *J. R. Soc. Interface* 9, 110-118. 10.1098/rsif.2011.0182 21613286 PMC3223624

[JEB252394C26] Fukunaga, T., Roy, R. R., Shellock, F. G., Hodgson, J. A. and Edgerton, V. R. (1996). Specific tension of human plantar flexors and dorsiflexors. *J. Appl. Physiol. Bethesda Md. 1985* 80, 158-165. 10.1152/jappl.1996.80.1.158 8847297

[JEB252394C27] Golyski, P. R. and Sawicki, G. S. (2022). Which lower limb joints compensate for destabilizing energy during walking in humans? *J. R. Soc. Interface* 19, 20220024. 10.1098/rsif.2022.0024 35642426 PMC9156907

[JEB252394C28] Handsfield, G. G., Meyer, C. H., Hart, J. M., Abel, M. F. and Blemker, S. S. (2014). Relationships of 35 lower limb muscles to height and body mass quantified using MRI. *J. Biomech.* 47, 631-638. 10.1016/j.jbiomech.2013.12.002 24368144

[JEB252394C29] Herzog, W., Abrahamse, S. K. and ter Keurs, H. E. (1990). Theoretical determination of force-length relations of intact human skeletal muscles using the cross-bridge model. *Pflugers Arch.* 416, 113-119. 10.1007/BF00370231 2352828

[JEB252394C30] Jacobs, R., Bobbert, M. F. and van Ingen Schenau, G. J. (1996). Mechanical output from individual muscles during explosive leg extensions: The role of biarticular muscles. *J. Biomech.* 29, 513-523. 10.1016/0021-9290(95)00067-4 8964781

[JEB252394C31] Jakubowski, K. L., Martino, G., Beck, O. N., Sawicki, G. S. and Ting, L. H. (2025). Center of mass states render multijoint torques throughout standing balance recovery. *J. Neurophysiol.* 133, 206-221. 10.1152/jn.00367.2024 39658948 PMC11967846

[JEB252394C32] Johnson, M. A., Polgar, J., Weightman, D. and Appleton, D. (1973). Data on the distribution of fibre types in thirty-six human muscles. An autopsy study. *J. Neurol. Sci.* 18, 111-129. 10.1016/0022-510X(73)90023-3 4120482

[JEB252394C33] Ker, R. F. (1981). Dynamic tensile properties of the plantaris tendon of sheep (*Ovis aries*). *J. Exp. Biol.* 93, 283-302. 10.1242/jeb.93.1.283 7288354

[JEB252394C34] Kharazi, M., Bohm, S., Theodorakis, C., Mersmann, F. and Arampatzis, A. (2021). Quantifying mechanical loading and elastic strain energy of the human Achilles tendon during walking and running. *Sci. Rep.* 11, 5830. 10.1038/s41598-021-84847-w 33712639 PMC7955091

[JEB252394C35] Kharazi, M., Theodorakis, C., Mersmann, F., Bohm, S. and Arampatzis, A. (2023). Contractile work of the soleus and biarticular mechanisms of the gastrocnemii muscles increase the net ankle mechanical work at high walking speeds. *Biology* 12, 872. 10.3390/biology12060872 37372156 PMC10295290

[JEB252394C36] Klein, P., Mattys, S. and Rooze, M. (1996). Moment arm length variations of selected muscles acting on talocrural and subtalar joints during movement: an in vitro study. *J. Biomech.* 29, 21-30. 10.1016/0021-9290(95)00025-9 8839014

[JEB252394C37] Konow, N., Azizi, E. and Roberts, T. J. (2012). Muscle power attenuation by tendon during energy dissipation. *Proc. R. Soc. B Biol. Sci.* 279, 1108-1113. 10.1098/rspb.2011.1435 PMC3267137 21957134

[JEB252394C38] Li, W., Keegan, T. H. M., Sternfeld, B., Sidney, S., Quesenberry, C. P. and Kelsey, J. L. (2006). Outdoor falls among middle-aged and older adults: a neglected public health problem. *Am. J. Public Health* 96, 1192-1200. 10.2105/AJPH.2005.083055 16735616 PMC1483851

[JEB252394C39] Mademli, L., Arampatzis, A., Morey-Klapsing, G. and Brüggemann, G.-P. (2004). Effect of ankle joint position and electrode placement on the estimation of the antagonistic moment during maximal plantarflexion. *J. Electromyogr. Kinesiol. Off. J. Int. Soc. Electrophysiol. Kinesiol.* 14, 591-597. 10.1016/j.jelekin.2004.03.006 15301777

[JEB252394C40] Maiwald, C., Sterzing, T., Mayer, T. A. and Milani, T. L. (2009). Detecting foot-to-ground contact from kinematic data in running. *Footwear Sci.* 1, 111-118. 10.1080/19424280903133938

[JEB252394C41] Mersmann, F., Bohm, S., Schroll, A. and Arampatzis, A. (2014). Validation of a simplified method for muscle volume assessment. *J. Biomech.* 47, 1348-1352. 10.1016/j.jbiomech.2014.02.007 24607005

[JEB252394C42] Mersmann, F., Bohm, S., Schroll, A., Boeth, H., Duda, G. and Arampatzis, A. (2015). Muscle shape consistency and muscle volume prediction of thigh muscles. *Scand. J. Med. Sci. Sports* 25, e208-e213. 10.1111/sms.12285 24975992

[JEB252394C43] Nakazawa, K., Kawashima, N., Akai, M. and Yano, H. (2004). On the reflex coactivation of ankle flexor and extensor muscles induced by a sudden drop of support surface during walking in humans. *J. Appl. Physiol.* 96, 604-611. 10.1152/japplphysiol.00670.2003 14527965

[JEB252394C44] Neptune, R. R., Sasaki, K. and Kautz, S. A. (2008). The effect of walking speed on muscle function and mechanical energetics. *Gait Posture* 28, 135-143. 10.1016/j.gaitpost.2007.11.004 18158246 PMC2409271

[JEB252394C45] Pandy, M. G., Lin, Y.-C. and Kim, H. J. (2010). Muscle coordination of mediolateral balance in normal walking. *J. Biomech.* 43, 2055-2064. 10.1016/j.jbiomech.2010.04.010 20451911

[JEB252394C46] Pijnappels, M., Bobbert, M. F. and van Dieën, J. H. (2005). Push-off reactions in recovery after tripping discriminate young subjects, older non-fallers and older fallers. *Gait Posture* 21, 388-394. 10.1016/j.gaitpost.2004.04.009 15886128

[JEB252394C47] Prilutsky, B. I. and Zatsiorsky, V. M. (1994). Tendon action of two-joint muscles: transfer of mechanical energy between joints during jumping, landing, and running. *J. Biomech.* 27, 25-34. 10.1016/0021-9290(94)90029-9 8106533

[JEB252394C48] Prilutsky, B. I., Herzog, W. and Leonard, T. (1996). Transfer of mechanical energy between ankle and knee joints by gastrocnemius and plantaris muscles during cat locomotion. *J. Biomech.* 29, 391-403. 10.1016/0021-9290(95)00054-2 8964769

[JEB252394C49] Roberts, T. J. and Konow, N. (2013). How tendons buffer energy dissipation by muscle. *Exerc. Sport Sci. Rev.* 41, 186-193. 10.1097/JES.0b013e3182a4e6d5 23873133 PMC3836820

[JEB252394C50] Schulze, F., Mersmann, F., Bohm, S. and Arampatzis, A. (2012). A wide number of trials is required to achieve acceptable reliability for measurement patellar tendon elongation in vivo. *Gait Posture* 35, 334-338. 10.1016/j.gaitpost.2011.09.107 22178032

[JEB252394C51] Schumacher, C., Berry, A., Lemus, D., Rode, C., Seyfarth, A. and Vallery, H. (2019). Biarticular muscles are most responsive to upper-body pitch perturbations in human standing. *Sci. Rep.* 10, 14492. 10.1038/s41598-019-50995-3 PMC6787002 31601860

[JEB252394C52] Schumacher, C., Sharbafi, M., Seyfarth, A. and Rode, C. (2020). Biarticular muscles in light of template models, experiments and robotics: a review. *J. R. Soc. Interface* 17, 20180413. 10.1098/rsif.2018.0413 32093540 PMC7061696

[JEB252394C53] Sharbafi, M. A., Rode, C., Kurowski, S., Scholz, D., Möckel, R., Radkhah, K., Zhao, G., Mohammadinejad Rashty, A., von Stryk, O. and Seyfarth, A. (2016). A new biarticular actuator design facilitates control of leg function inBioBiped3. *Bioinspir. Biomim.* 11, 046003. 10.1088/1748-3190/11/4/046003 27367459

[JEB252394C54] Sharbafi, M. A., Mohammadi, A., Rashty, N., Rode, C. and Seyfarth, A. (2017). Reconstruction of human swing leg motion with passive biarticular muscle model. *Hum. Mov. Sci.* 52, 96-107. 10.1016/j.humov.2017.01.008 28182970

[JEB252394C55] Spoor, C. W., van Leeuwen, J. L., Meskers, C. G., Titulaer, A. F. and Huson, A. (1990). Estimation of instantaneous moment arms of lower-leg muscles. *J. Biomech.* 23, 1247-1259. 10.1016/0021-9290(90)90382-D 2292604

[JEB252394C56] Theodorakis, C., Bohm, S., Epro, G., Mersmann, F., Werth, J., Karamanidis, K. and Arampatzis, A. (2025). Enhanced joint energy transfer potential by the biarticular gastrocnemii muscles during perturbed walking. *Eur. J. Appl. Physiol.* 125, 2117-2131. 10.1007/s00421-025-05727-z 40042657 PMC12354497

[JEB252394C57] Van Der Linden, M. H., Marigold, D. S., Gabreëls, F. J. M. and Duysens, J. (2007). Muscle Reflexes and Synergies Triggered by an Unexpected Support Surface Height During Walking. *J. Neurophysiol.* 97, 3639-3650. 10.1152/jn.01272.2006 17392408

[JEB252394C58] van Dieën, J. H., Spanjaard, M., Konemann, R., Bron, L. and Pijnappels, M. (2007). Balance control in stepping down expected and unexpected level changes. *J. Biomech.* 40, 3641-3649. 10.1016/j.jbiomech.2007.06.009 17644100

[JEB252394C59] Van Ingen Schenau, G. J. (1989). From rotation to translation: Constraints on multi-joint movements and the unique action of bi-articular muscles. *Hum. Mov. Sci.* 8, 301-337. 10.1016/0167-9457(89)90037-7

[JEB252394C60] Wade, L., Lichtwark, G. and Farris, D. (2024). Implementation of a passive bi-articular ankle-knee exoskeleton during maximal squat jumping. *R. Soc. Open Sci.* 11, 240390. 10.1098/rsos.240390 39086826 PMC11288684

[JEB252394C61] Werkhausen, A., Albracht, K., Cronin, N. J., Meier, R., Bojsen-Møller, J. and Seynnes, O. R. (2017). Modulation of muscle–tendon interaction in the human triceps surae during an energy dissipation task. *J. Exp. Biol.* 220, 4141-4149. 10.1242/jeb.164111 28883087

[JEB252394C62] Zajac, F. E. (1989). Muscle and tendon: properties, models, scaling, and application to biomechanics and motor control. *Crit. Rev. Biomed. Eng.* 17, 359-411. 2676342

